# Selectome update: quality control and computational improvements to a database of positive selection

**DOI:** 10.1093/nar/gkt1065

**Published:** 2013-11-12

**Authors:** Sébastien Moretti, Balazs Laurenczy, Walid H. Gharib, Briséïs Castella, Arnold Kuzniar, Hannes Schabauer, Romain A. Studer, Mario Valle, Nicolas Salamin, Heinz Stockinger, Marc Robinson-Rechavi

**Affiliations:** ^1^Department of Ecology and Evolution, Biophore, University of Lausanne, CH-1015 Lausanne, Switzerland, ^2^Evolutionary Bioinformatics group, SIB Swiss Institute of Bioinformatics, CH-1015 Lausanne, Switzerland, ^3^Vital-IT group, SIB Swiss Institute of Bioinformatics, CH-1015 Lausanne, Switzerland, ^4^Computational Phylogenetics group, SIB Swiss Institute of Bioinformatics, CH-1015 Lausanne, Switzerland, ^5^Division of Biosciences, Institute of Structural and Molecular Biology, University College London, Gower Street, London, WC1E 6BT, UK and ^6^Swiss National Supercomputing Centre (CSCS), CH-6900, Lugano, Switzerland

## Abstract

Selectome (http://selectome.unil.ch/) is a database of positive selection, based on a branch-site likelihood test. This model estimates the number of nonsynonymous substitutions (dN) and synonymous substitutions (dS) to evaluate the variation in selective pressure (dN/dS ratio) over branches and over sites. Since the original release of Selectome, we have benchmarked and implemented a thorough quality control procedure on multiple sequence alignments, aiming to provide minimum false-positive results. We have also improved the computational efficiency of the branch-site test implementation, allowing larger data sets and more frequent updates. Release 6 of Selectome includes all gene trees from Ensembl for Primates and Glires, as well as a large set of vertebrate gene trees. A total of 6810 gene trees have some evidence of positive selection. Finally, the web interface has been improved to be more responsive and to facilitate searches and browsing.

## INTRODUCTION

Selectome is a database of positive selection (1). It provides users with access to precomputed estimates of positive selection from the branch-site test (2) mapped to branches of gene trees (including speciations and duplications), and to amino-acid sites of multiple sequence alignments (MSAs). This allows the detection of episodic selection, which is an important component of protein evolution (3). Selectome's first release was based on TreeFam A (PLACEHOLDER FOR NAR DATABASE UPDATE). While this choice was made to ensure high quality, it posed two problems: one is that TreeFam A was, by design, incomplete, and the other is that TreeFam has not been regularly updated. We have thus decided to move to Ensembl Compara (4) to receive gene trees and MSAs. Ensembl Compara provides a set of gene trees and MSAs as complete as possible, updated with every release of Ensembl (5). Moreover, using Ensembl's gene trees and MSAs allow easy extension to other taxonomic groups, which are covered by the Ensembl Genomes projects (6).

The transition from TreeFam A to TreeFam A + B then to Ensembl Compara has raised two major challenges: (i) computing branch-site positive selection (2) on hundreds of thousands of branches from thousands of gene trees is a major computational challenge, especially considering that CodeML from PAML (7) has never been optimized with respect to computational efficiency; (ii) the MSAs provided by the automated Compara pipeline, while sufficient for many purposes, contain many misaligned regions, which induce false positives in tests for positive selection, especially for the branch-site test (8–10) (the same is true of other pipelines). These false-positive issues led us to label ‘*beta*’ several releases after the transition away from TreeFam A.

We present the latest release of Selectome (release 6), which is the first release based on Ensembl Compara to take advantage of improvements concerning both computational efficiency and MSA quality control.

## CHANGES IN DATABASE CONTENT

**Figure 1.**
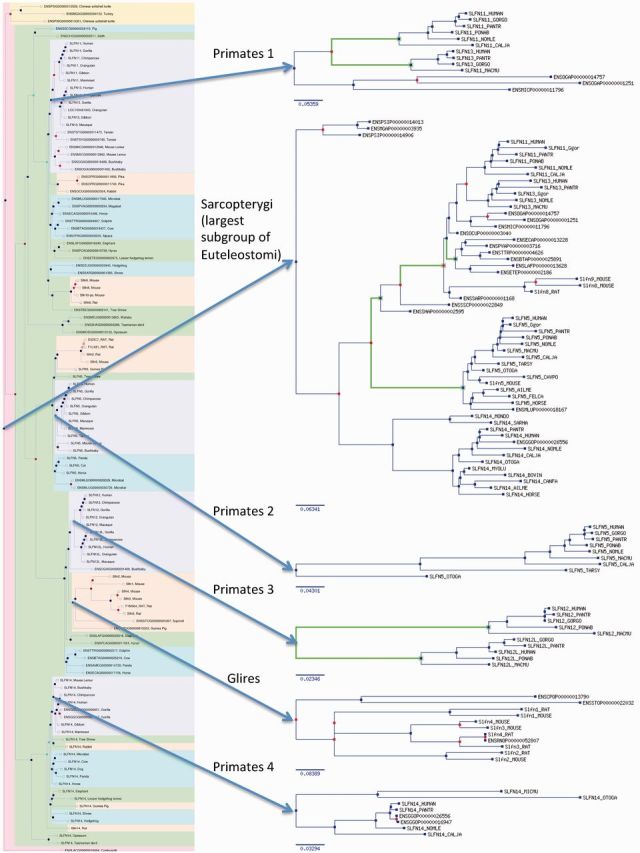
Selectome subtrees from Ensembl Compara gene tree. Left, the tree for human gene ENSGT00410000025651 from Ensembl release 68. Right, the subtrees selected for use in Selectome. Note that (i) as the tree is rooted in *Amniota* (i.e. there are no homologs detected outside *Amniota*), which is a subset of *Euteleostomi*, this node was chosen for the subtree for *Euteleostomi*; (ii) there are four *Primate* subtrees, due to gene duplications; (iii) only the *Glires* subtree with at least six sequences was used; (iv) some *Primate* or *Glires* subtrees can differ from the Ensembl tree because they use later Ensembl releases (Table 1).

A summary of the content of Selectome release 6 is presented in Table 1. We define taxon-specific subtrees as monophyletic groups, which contain only sequences from the target taxon (Figure 1). We have computed branch-site tests for positive selection for all internal branches of all gene trees of Primates and of Glires, which contained at least six sequences (leaves of the subtree) after alignment quality control. We have also computed the tests for all internal branches of small- to medium-sized gene trees, which cover all Euteleostomi. As in previous releases of Selectome (1), multiple testing is controlled with a q-value of 10% computed over the union of all test results (all branches, all trees); this was done separately for each taxonomic group (i.e. Primates, Glires, Euteleostomi).

**Table 1.** Statistics on release 06 of Selectome

Taxonomic group	Species number	Ensembl release	Subtrees^a^				Sequences per subtree	
			Total	Filtered^b^	Computed	With positive selection	Median	Max
Euteleostomi	54	68	19 940	15 923	13 695^c^	6543	32	139
Glires	7	71	20 114	4656^d^	4656	136	6	257
Primates	10	70	20 300	15 738	15 738	131	8	180

^a^Pruned from larger Ensembl Compara trees, according to the taxonomic group.

^b^Subtrees with at least six sequences after alignment quality filtering.

^c^The largest gene trees were not computed.

^d^Many Glires subtrees do not have six sequences before or after our filtering.

Since Selectome is now based on Ensembl, all cross-references, taxonomic information, keywords, and other information are now from Ensembl, and no longer from TreeFam.

We have first tackled the computational challenge of updating Selectome by a better use of computing infrastructure. CodeML has been ported to the Swiss multi-scientific computing grid SMSCG (http://www.smscg.ch). All computations for Primates data were done on this infrastructure, using a customized GC3pie framework (11), which notably manages submissions and error messages. We experienced a failure rate of 0.7%, i.e. submission/execution issues that are due to the Grid infrastructure (including exceeding allocated execution time for single jobs). All erroneous jobs were successfully resubmitted. Thus, 67 054 job pairs (H0 and H1 hypotheses of the test sequentially on the same node) were successfully computed on SMSCG, and 276 were computed on the Vital-IT computer cluster (http://www.vital-it.ch), because they exceeded the runtime limit of SMSCG.

Secondly, we have optimized CodeML for the branch-site test. Briefly, SlimCodeML (12) is an optimized sequential version of CodeML, which provides identical results to the original code. All computations for Euteleostomi and Glires were performed using SlimCodeML on the Vital-IT cluster. For Euteleostomi, the 2228 largest subtrees were not computed because of time limitations on the cluster. This showed again an intrinsic performance/scalability problem of (Slim)CodeML with respect to large data sets.

In the original Selectome pipeline, poorly aligned regions were removed using GBLOCKS (13), but both our experience and published benchmarks (8–10,14) indicate that this is insufficient to remove unreliable regions of MSAs, which cause false positives for the branch-site test of positive selection. The Selectome pipeline now includes the following: realignment with PAGAN (15); masking of amino-acids that have a low consistency score from M-Coffee (16); and masking of amino-acids that have a low score from GUIDANCE (17). In addition, MaxAlign (18) is used to remove sequences that have few unambiguous sites, relative to the rest of the alignment, and TrimAl (19) is used to remove columns with few unambiguous sites. Detailed procedures and thresholds for each release are provided at http://selectome.unil.ch/cgi-bin/methods.cgi. Of note, Privman *et al.* (14) showed that the loss of true positives by filtering was outweighed by the removal of false positives. In total, 8.7% of MSA columns were removed before selection computations for Primates, versus 4.4% in Selectome 5 (GBLOCKS based pipeline); 12% of columns were removed for Glires, and 34% of columns for Euteleostomi, consistent with the expectation that more divergent sequences are more difficult to align reliably. More in detail, in Selectome 5, in Primates we identified 246 678 out of 1 149 639 sites (21%) as under positive selection, including long continuous stretches of ‘positively selected’ sites, which manual examination showed to be alignment or gene model errors [consistent with (10)]. In Selectome 6, filtering reduced the number of sites analyzed to 392 104, of which 61 119 are identified as under positive selection (16%); there are no more long stretches of sites, and manual inspection does not identify any obvious false positives. Further benchmarking of this pipeline shows that it masks not only MSA regions, which are difficult to align because of low complexity or alignment heuristics, but also gene model errors, which are a major source of false positives in MSAs from genomics (Moretti and Robinson-Rechavi, in preparation). By gene model errors, we mean errors in exon boundaries, in coding sequence start or stop, in prediction or choice of transcript from the gene; all these can lead to the alignment of nonhomologous sites.

MSAs, which have less than six sequences or no aligned columns left after the filtering pipeline are not included in Selectome; this is notably the case for many Glires subtrees (Table 1).

## CHANGES IN WEB INTERFACE

The Selectome web interface is similar to the original TreeFam interface, but with specific enrichments. We list here the main improvements of the interface since Selectome release 1.

Improved search: For keyword search, queries are faster, thanks to the use of Sphinx (http://sphinxsearch.com), and queries are automatically restricted to the most relevant field (e.g. gene, species, cross-reference), which can then be manually modified. For advanced search, a species tree of interest can be chosen (i.e. Euteleostomi, Primates, Glires). Query results can now be viewed by genes or by gene families (subtrees), and sorting is possible according to each column (e.g. selection, taxon, gene name). Moreover, results can be filtered by species or keyword.

Improved graphical user interface: Each query result includes a preview of the gene tree with selection highlighted. On the gene family (subtree) view, positive selection is now indicated by a highlight of the whole branch, rather than a discrete box on the node; there is easy navigation between subtrees from the same Ensembl family; and it is possible to change the size of the gene tree image. For MSA visualization (with the annotation of detected sites under positive selection) in Jalview (20), unreliably aligned sites (not used for computation) can be masked (indicated by the character ‘x’). Finally, we provide a DAS service (http://selectome.unil.ch/das/selectome) for integration with other resources [distributed annotation system (21)]. Selectome is also indexed and searchable by the ExPASy portal (http://expasy.org/), and external links to Ensembl point toward the version of Ensembl used for each result to ensure consistency; of note, linking to specific versions is not yet possible for Ensembl Genomes.

## CONCLUSIONS AND PERSPECTIVES

Selectome presents, to our knowledge, the only phylogenomic database of branch-site positive selection (discussion of other resources in 1). The most significant progress since the first release is the improved MSA filtering, which dramatically reduces false positives, and allows us to use different input sources: if the input includes low-quality sequences, gene or transcript models or alignments, they are not used for positive selection inference. The use of Ensembl and the improved computational efficiency allow us to present for the first time a database with complete computations of branch-site positive selection for the two most studied mammalian clades: Primates, Glires. The next release of Selectome will also include the *Drosophila* clade.

The major future challenge of Selectome is to further increase computational efficiency, to allow complete computations on large clades such as vertebrates (Euteleostomi), arthropods or green plants. The use of Ensembl and the existence of the Ensembl Genomes projects provide consistent data sources for most clades of interest. We have recently confirmed that the branch-site test can be reliably used even on deep nodes of such clades (22); the results of our partial release on Euteleostomi moreover confirm that with these larger gene trees, we have satisfactory power to detect positive selection (Table 1). The proportion of Euteleostomi genes with positive selection (48%) is lower than the 77% reported previously on a smaller sample (23) (biased toward genes conserved among vertebrates), but remains high, and should be further investigated. A potential problem, which we have not yet addressed, is synonymous rate variation between sites (24), which has been shown to be a problem for the site-test but has not been investigated for the branch-site test. As methods of detecting episodic positive selection improve, they will be taken into account in Selectome.

Given the runtime issues for large data sets, we have developed a new, parallel and highly optimized software for the branch-site model: FastCodeML (Valle *et al.*, in preparation; ftp://ftp.vital-it.ch/tools/FastCodeML/). Tests show that running this software on a supercomputer allows computing positive selection even on the largest Ensembl Compara gene trees. Future Selectome releases will thus use FastCodeML on a mixture of commodity computers as well as large cluster computer systems and eventually computational grids. Our aim is to provide yearly updates that cover Ensembl-type data as completely as possible, given the constraints on MSA quality.

## FUNDING

Project UNIL.5 (Grid/Selectome) of the ‘AAA/SWITCH–e-infrastructure for e-science’ program; the Swiss Platform for High-Performance and High-Productivity Computing (HP2C); the Swiss National Science Foundation [31003A 133011/1 to M.R.R. and CR32I3_143768 to N.S. and M.R.R.]; Etat de Vaud; Fondation du 450ème anniversaire de l'Université de Lausanne and Swiss National Science Foundation [132476 and 136477 to R.A.S.]. Cluster computations were performed at the Vital-IT (http://www.vital-it.ch) Center for high-performance computing of the SIB Swiss Institute of Bioinformatics. Funding for open access charge: Etat de Vaud.

*Conflict of interest statement*. None declared.

## References

[1] Proux E, Studer RA, Moretti S, Robinson-Rechavi M (2009). Selectome: a database of positive selection. Nucleic Acids Res..

[2] Zhang J, Nielsen R, Yang Z (2005). Evaluation of an improved branch-site likelihood method for detecting positive selection at the molecular level. Mol. Biol. Evol..

[3] Studer RA, Robinson-Rechavi M (2009). Evidence for an episodic model of protein sequence evolution. Biochem. Soc. Trans..

[4] Vilella AJ, Severin J, Ureta-Vidal A, Heng L, Durbin R, Birney E (2009). EnsemblCompara GeneTrees: complete, duplication-aware phylogenetic trees in vertebrates. Genome Res..

[5] Flicek P, Ahmed I, Amode MR, Barrell D, Beal K, Brent S, Carvalho-Silva D, Clapham P, Coates G, Fairley S (2013). Ensembl 2013. Nucleic Acids Res.

[6] Kersey PJ, Staines DM, Lawson D, Kulesha E, Derwent P, Humphrey JC, Hughes DS, Keenan S, Kerhornou A, Koscielny G (2012). Ensembl Genomes: an integrative resource for genome-scale data from non-vertebrate species. Nucleic Acids Res..

[7] Yang Z (2007). PAML 4: phylogenetic analysis by maximum likelihood. Mol. Biol. Evol..

[8] Fletcher W, Yang Z (2010). The effect of insertions, deletions and alignment errors on the branch-site test of positive selection. Mol. Biol. Evol..

[9] Jordan G, Goldman N (2012). The effects of alignment error and alignment filtering on the sitewise detection of positive selection. Mol. Biol. Evol..

[10] Markova-Raina P, Petrov D (2011). High sensitivity to aligner and high rate of false positives in the estimates of positive selection in the 12 Drosophila genomes. Genome Res..

[11] Moretti S, Murri R, Maffioletti S, Kuzniar A, Castella B, Salamin N, Robinson-Rechavi M, Stockinger H (2012). gcodeml: a grid-enabled tool for detecting positive selection in biological evolution. Stud. Health Technol. Inform..

[12] Schabauer H, Valle M, Pacher C, Stockinger H, Stamatakis A, Robinson-Rechavi M, Yang Z, Salamin N (2012). SlimCodeML: an optimized version of CodeML for the branch-site model. In: IEEE International Workshop on High Performance Computational Biology (HiCOMB’12) Shanghai.

[13] Talavera G, Castresana J (2007). Improvement of phylogenies after removing divergent and ambiguously aligned blocks from protein sequence alignments. Syst. Biol..

[14] Privman E, Penn O, Pupko T (2012). Improving the performance of positive selection inference by filtering unreliable alignment regions. Mol. Biol. Evol..

[15] Loytynoja A, Vilella AJ, Goldman N (2012). Accurate extension of multiple sequence alignments using a phylogeny-aware graph algorithm. Bioinformatics.

[16] Wallace IM, O'Sullivan O, Higgins DG, Notredame C (2006). M-Coffee: combining multiple sequence alignment methods with T-Coffee. Nucleic Acids Res..

[17] Penn O, Privman E, Landan G, Graur D, Pupko T (2010). An alignment confidence score capturing robustness to guide-tree uncertainty. Mol. Biol. Evol..

[18] Gouveia-Oliveira R, Sackett P, Pedersen A (2007). MaxAlign: maximizing usable data in an alignment. BMC Bioinformatics.

[19] Capella-Gutiérrez S, Silla-Martínez JM, Gabaldón T (2009). trimAl: a tool for automated alignment trimming in large-scale phylogenetic analyses. Bioinformatics.

[20] Waterhouse AM, Procter JB, Martin DM, Clamp M, Barton GJ (2009). Jalview Version 2—a multiple sequence alignment editor and analysis workbench. Bioinformatics.

[21] Dowell R, Jokerst R, Day A, Eddy S, Stein L (2001). The distributed annotation system. BMC Bioinformatics.

[22] Gharib WH, Robinson-Rechavi M (2013). The branch-site test of positive selection is surprisingly robust but lacks power under synonymous substitution saturation and variation in GC. Mol. Biol. Evol..

[23] Studer RA, Penel S, Duret L, Robinson-Rechavi M (2008). Pervasive positive selection on duplicated and nonduplicated vertebrate protein coding genes. Genome Res..

[24] Rubinstein ND, Doron-Faigenboim A, Mayrose I, Pupko T (2011). Evolutionary models accounting for layers of selection in protein-coding genes and their impact on the inference of positive selection. Mol. Biol. Evol..

